# The draft genome sequence of the ascomycete fungus *Colletotrichum acutatum* obtained from strawberry roots

**DOI:** 10.1128/mra.01357-22

**Published:** 2026-04-01

**Authors:** Moytri RoyChowdhury, Jake Sternhagen, Yookyeong Kim

**Affiliations:** 1 California Department of Public Health, Infectious Diseases Program 663764 , Richmond, California, USA; 2 Department of Biological Sciences, Idaho State University 6640, Pocatello, Idaho, USA; 3 Riverside School of Medicine; SOM Education Building, University of California 378743 , Riverside, California, USA; 4 UC Berkeley Extensions, Berkeley, California, USA; University of California Riverside, Riverside, California, USA

**Keywords:** fungi, genome analysis, genome sequencing, ascomycetes

## Abstract

Here we report the genome sequence of the ascomycete pathogenic fungus *Colletotrichum acutatum* isolate #83 obtained from the rootstock of strawberries in Santa Maria, CA.

## ANNOUNCEMENT

*Colletotrichum acutatum* causes severe economic losses in the production of strawberries in California (1). The absence of genomic sequences of *C. acutatum* is a major hindrance in understanding the development and pathogenicity of this fungus, so this sequence information will facilitate future research into this key pathogen. Here we present the whole genome sequence of the *C. acutatum* isolate #83 obtained from the rootstock (underground stem/rhizome that can produce aboveground growth) of strawberries in Santa Maria, CA.

The fungus was grown from a pure-culture, strawberry rootstock-derived isolate #83 of *C. acutatum* was provided by Plant Sciences, Inc., Watsonville, CA, USA. A 0.6 cm diameter plug of developed hyphae was placed in the center of 3.9% potato dextrose agar (manufactured by Millipore Sigma P-2182) plates and grown in the dark at 27°C for 15 days.

In the DNA extraction, 15-day-old mycelium was used to extract DNA from the fungal isolates using the E.Z.N.A. Universal Pathogen Kit (Omega Biotek) with additional modifications for a more robust mechanical lysis with the OMNI disruptor, three cycles of freeze and thaw, and a hot phenol extraction. DNA was quantified using PicoGreen, obtaining 400 µL at approximately 10 ng/µL for a total of 4 µg of DNA. DNA concentration was measured using the QuantiFluor dsDNA System on a Quantus Fluorometer (Promega, Madison, WI, USA).

For the Illumina library prep, a Kapa Biosystems Hyper Prep kit (Kapa Biosystems, Wilmington, MA, USA) was used for whole genome library construction. One microgram of genomic DNA was fragmented using a Bioruptor sonicator (Diagenode, Denville, NJ, USA). DNA fragment ends were repaired, 3′ adenylated, and ligated to adapters. The resulting adapter-ligated libraries were PCR-amplified to create enough genetic material for sequencing. Illumina indexes were added and pooled for multiplexed sequencing on an Illumina HiSeq 2500 sequencer (Illumina, San Diego, CA, USA) using the paired-end 100 bp run format.

Sequencing statistics showed that a total of 12,447,195 paired reads were generated by Illumina sequencing that was estimated as a 17× fold coverage of the genome. FastQC was used to determine read quality, and it did not flag any sequencing reads as poor quality and estimated the GC content as 50%. The reads were filtered using Trimmomatic (v.0.30) (2) to remove primers, adapter sequences, and low-quality bases using default settings. The genome assembly was performed with SPAdes (v.3.10.0, 27 January 2017) (JAJKBH000000000). The genome assembly length was 49,743,065 bp and consisted of 1,998 contigs with an NGA50 of 57,457 bp (Table 1), as computed by QUAST-LG (3, 4). The reference genome that was compared to the alignment as part of the QUAST-LG run was *Colletotrichum scovillei* strain (PRJNA314171) (5).

**TABLE 1:** Genome sequence of *C. acutatum* W537^*a*^

Statistics	Results
Genome statistics (contigs)	
Genome fraction (%)	77.192
Duplication ratio	1.001
Largest alignment	342,153
Total aligned length	40,233,184
NGA50	57,457
LGA50	232
Misassemblies	
No. of misassemblies	264
Length of misassembled contigs	24,608,249
Mismatches	
No. of mismatches/100 kbp	2,646.08
No. of indels/100 kbp	236.71
No. of N’s/100 kbp	0
Statistics without reference	
No. of contigs	1,172
Largest contig	909,718
Total length	49,743,065
Total length (≥1,000 bp)	49,530,365
Total length (≥10,000 bp)	48,498,476
Total length (≥50,000 bp)	42,041,346

^*a*^ Regarding all software, default parameters were used except where otherwise noted.

NGA50 are analogous to N50 and NG50 where the contigs are replaced by blocks that can be aligned to the reference. Correctness is measured by detecting misassemblies such as mismatches, indels, and misjoins (6). Our analysis pipeline at the time of sequencing this sample did not include BUSCO assessments.

## Data Availability

This Whole Genome Shotgun project has been deposited in GenBank (accession no. JAJKBH000000000 and SRA accession no. SRR16974093). The version described in this paper is the first version (JAJKBH010000000, BioProject no. PRJNA779114). The data of the reference genome have been deposited with links to BioProject accession number PRJNA314171 in the NCBI BioProject database (https://www.ncbi.nlm.nih.gov/bioproject/).
